# Correction: Widespread Hypomethylation Occurs Early and Synergizes with Gene Amplification during Esophageal Carcinogenesis

**DOI:** 10.1371/annotation/8dcded85-a924-40f4-a7ea-56961b87447f

**Published:** 2011-05-06

**Authors:** Hector Alvarez, Joanna Opalinska, Li Zhou, Davendra Sohal, Melissa J. Fazzari, Yiting Yu, Christina Montagna, Elizabeth A. Montgomery, Marcia Canto, Kerry B. Dunbar, Jean Wang, Juan Carlos Roa, Yongkai Mo, Tushar Bhagat, K. H. Ramesh, Linda Cannizzaro, J. Mollenhauer, Reid F. Thompson, Masako Suzuki, Stephen Meltzer, Ari Melnick, John M. Greally, Anirban Maitra, Amit Verma

Stephen Meltzer's middle initial was omitted in the published article. His name should be: Stephen J. Meltzer.

